# Age-related sensitivity to endotoxin-induced liver inflammation: Implication of inflammasome/IL-1β for steatohepatitis

**DOI:** 10.1111/acel.12305

**Published:** 2015-04-07

**Authors:** Ki Wung Chung, Eun Kyeong Lee, Dae Hyun Kim, Hye Jin An, Nam Deuk Kim, Dong Soon Im, Jaewon Lee, Byung Pal Yu, Hae Young Chung

**Affiliations:** 1 Molecular Inflammation Research Center for Aging Intervention (MRCA), College of Pharmacy, Pusan National UniversityBusan, Korea; 2 Department of Physiology, The University of Texas Health Science Center at San AntonioSan Antonio, TX, 78229-3900, USA

**Keywords:** aging, IL-1β, inflammasome, inflammation, lipid accumulation, LPS

## Abstract

Aging is associated with increased vulnerability to inflammatory challenge. However, the effects of altered inflammatory response on the metabolic status of tissues or organs are not well documented. In this study, we present evidence demonstrating that lipopolysaccharide (LPS)-induced upregulation of the inflammasome/IL-1β pathway is accompanied with an increased inflammatory response and abnormal lipid accumulation in livers of aged rats. To monitor the effects of aging on LPS-induced inflammation, we administered LPS (2 mg kg^−1^) to young (6-month old) and aged (24-month old) rats and found abnormal lipid metabolism in only aged rats with increased lipid accumulation in the liver. This lipid accumulation in the liver was due to the dysregulation of PPARα and SREBP1c. We also observed severe liver inflammation in aged rats as indicated by increased ALT levels in serum and increased Kupffer cells in the liver. Importantly, among many inflammation-associated factors, the aged rat liver showed chronically increased IL-1β production. Increased levels of IL-1β were caused by the upregulation of caspase-1 activity and inflammasome activation. *In vitro* studies with HepG2 cells demonstrated that treatment with IL-1β significantly induced lipid accumulation in hepatocytes through the regulation of PPARα and SREBP1c. In summary, we demonstrated that LPS-induced liver inflammation and lipid accumulation were associated with a chronically overactive inflammasome/IL-1β pathway in aged rat livers. Based on the present findings, we propose a mechanism of aging-associated progression of steatohepatitis induced by endotoxin, delineating a pathogenic role of the inflammasome/IL-1β pathway involved in lipid accumulation in the liver.

## Introduction

Nonalcoholic fatty liver disease (NAFLD) is one of the most common liver diseases and affects more than one-third of the population of the Western world (Vuppalanchi & Chalasani, 2009). The spectrum of NAFLD ranges from relatively benign hepatic steatosis characterized by the accumulation of triglycerides (TG) in hepatocytes to nonalcoholic steatohepatitis (NASH), which can progress to end-stage liver diseases such as cirrhosis and hepatocellular carcinoma (Wree *et al*., 2013). Although obesity, high-fat diets, and insulin resistance are recognized as risk factors for NAFLD (Wree *et al*., 2013), other significant factors that can lead to NAFLD remain to be identified. In addition to obesity, chronic inflammation is an important contributing factor in the pathogenesis of NASH (Tilg & Moschen, 2010).

Lipopolysaccharide (LPS), which is a major outer membrane component of gram-negative bacteria, also referred to as endotoxin, is considered a potent inducer of hepatic inflammation. LPS may be capable of stimulating inflammation, cytokine production, and accumulation of inflammatory cells within the liver (Affò *et al*., 2014). Indeed, intraperitoneal injection of a single dose of LPS accelerated hepatic lipid accumulation in animals (Chen *et al*., 2011). Several clinical investigations have revealed that, compared to the controls, serum LPS concentrations were increased in patients with NAFLD (Miele *et al*., 2009). In addition, several have reported that gut permeability, or small intestine bacterial overgrowth, was more frequently observed in NASH patients than in controls (Miele *et al*., 2009).

Aging is associated with an altered response to endotoxin-induced physiological changes, including inflammation and immune response (Saito *et al*., 2003). In older individuals, alterations of both innate and adaptive immunity lead to increased susceptibility to infections (Dorshkind *et al*., 2009). In particular, sepsis, which is an infection-initiated manifestation of a systemic inflammatory response syndrome, is common and important cause of increased mortality and morbidity in the elderly population (Gustot, 2011; Pinheiro da Silva *et al*., 2013). Animal models suggest that the pathophysiology of sepsis differs between young and aged animals. Compared with young animals, aged animals subjected to cecal ligation puncture (CLP) or endotoxemia have increased systemic levels of inflammatory cytokines (Turnbull *et al*., 2009). Although an altered innate immune system and exaggerated inflammation are major causes of increased morbidity following septic shock, it is important to elucidate the precise underlying mechanisms associated with these conditions.

While the effects of aging on the endotoxin-induced inflammatory response have been well studied, very little is known regarding the influence of inflammation on metabolism. As endotoxin-induced inflammation is known to affect whole-body energy metabolism including carbohydrate, lipid, and amino acid metabolism, a disruption in energetic metabolism is implicated in endotoxin-induced organ failure (Carré & Singer, 2008). As lipid metabolites play important roles as energy sources and signaling molecules, recent studies have focused on the effects of inflammation on changes of lipid metabolism (Glass & Olefsky, 2012). Various inflammatory signaling pathways are known to significantly alter lipid metabolism in the liver, adipose tissue, skeletal muscle, and macrophage in the context of infection, diabetes, and atherosclerosis (Glass & Olefsky, 2012). Indeed, endotoxin directly, or indirectly via cytokines, induces marked changes in lipid metabolism that are considered a part of the acute phase response (Feingold *et al*., 1992; Hardardóttir *et al*., 1994). These alterations can be beneficial as defense mechanisms, but prolonged changes can also be deleterious (Glass & Olefsky, 2012). In such instances, the liver is known to play an important role (Nesseler *et al*., 2012). It has been implicated in the host response, participating in the clearance of infectious agents and/or products, as well as mediating changes to lipid metabolism that occur during sepsis (Hardardóttir *et al*., 1994; Nesseler *et al*., 2012).

IL-1β is one of the principal inflammatory cytokines implicated in metabolic diseases, including diabetes (Febbraio, 2014). IL-1β is produced as inactive pro-IL-1β in response to inflammatory stimuli, including both microbial products and endogenous danger-associated molecules. Pro-IL-1β is then activated by complexes called inflammasomes (Rathinam et al., 2012). Inflammasomes are large caspase-1-activating multiprotein complexes that sense both exogenous and endogenous danger signals through intracellular NOD-like receptors (NLRs). IL-1β acts in an autocrine/paracrine manner via the type 1 IL-1 receptor. Emerging data have provided evidence for the role of IL-1 signaling in acute and chronic liver injury of diverse origins, including acetaminophen-induced liver damage (Imaeda *et al*., 2009), NASH (Miura *et al*., 2010), immune-mediated liver injury (Petrasek *et al*., 2011), and alcoholic steatohepatitis (Petrasek *et al*., 2012).

In a previous study, we reported that endotoxin induces higher activation of the NF-κB signaling pathway, especially in aged rat livers (Kwon *et al*., 2001). Here, we report that aging increases sensitivity to endotoxin-induced liver inflammation and lipid accumulation through the prolonged activation of inflammasomes and subsequent IL-1β production. We observed that endotoxin induces abnormal systemic accumulation of lipid metabolites and aged rat livers displayed increased lipid accumulation. Additionally, we found that the transcription factor peroxisome proliferator-activated receptor alpha (PPARα) was consistently inactivated and the levels of sterol regulatory element-binding transcription factor 1 (SREBP1c) were continuously increased in aged rat livers. Furthermore, we found that among the many inflammatory mediators, serum and liver levels of IL-1β were abnormally upregulated due to an increase in the caspase-1 activity of the inflammasome. Further analysis revealed the lipogenic role of IL-1β in hepatocytes. Based on these results, we propose a mechanism of aging-specific vulnerability against endotoxin-induced liver inflammation and suggest a pathogenic role of the inflammasome/IL-1β pathway in liver lipid accumulation.

## Results

### Aging increases sensitivity to LPS-induced lipid metabolism changes

**Fig 1:**
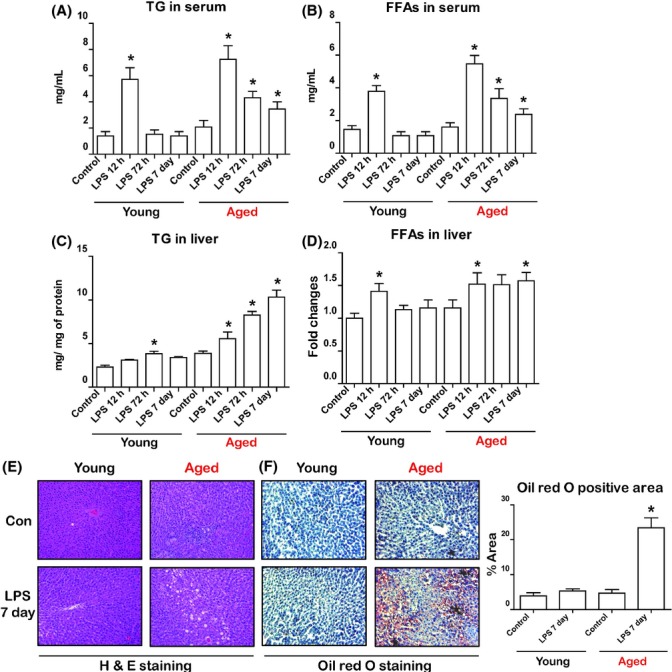
Aging increases sensitivity to lipopolysaccharide (LPS)-induced changes to lipid metabolism. Lipopolysaccharide was injected intraperitoneally (2 mg kg^−1^body weight) in young and aged rats. Rats were euthanized 12 h, 72 h, and 7 days after injection. (A) Measurement of triglycerides (TG) in serum following LPS injection in young and aged rats. (B) Measurement of free fatty acids (FFAs) in serum following LPS injection in young and aged rats. (C) Measurement of TG in the liver following LPS injection in young and aged rats. (D) Measurement of FFAs in the liver following LPS injection in young and aged rats. (E) Lipid accumulation in the livers was visualized by hematoxylin–eosin (H&E) staining. (F) Lipid accumulation in the livers was visualized by Oil red O staining. Oil red O positive areas were quantified. Data are expressed as the mean ± SEM. *P <* *0.05 in comparison with the corresponding controls.

Although the effects of endotoxin on lipid metabolism have been investigated (Feingold *et al*., 1992; Hardardóttir *et al*., 1994), the influence of aging on the process needs to be clarified. Therefore, to investigate whether aging influences endotoxin-induced changes to lipid metabolism, young (6 month) and aged (24 month) SD rats were injected intraperitoneally with LPS (2 mg kg^−1^) to mimic endotoxemia. To monitor the time-dependent effects, we euthanized rats 12 h, 72 h, and 7 days after LPS injection. The rats displayed no mortality during the endotoxemia and recovered from the symptoms of sepsis 72 h after injection. Serum levels of metabolites were measured to determine the effects of endotoxin on metabolic changes. Serum levels of TG and FFAs revealed an altered response in aged rats during endotoxemia (Fig.1). Compared to young rats, serum TG and FFAs of aged rats were continuously increased (Fig.1A,B). Total cholesterol levels also exhibited increase only in aged rats during endotoxemia (Fig. S1A, Supporting information). In contrast, serum glucose levels, also known to be altered during endotoxemia, were similar between young and aged rats (Fig. S1B).

Because the liver plays a pivotal role in the regulation of lipid metabolism during endotoxemia (Feingold *et al*., 1992), we next examined changes of lipid metabolism in the liver. Biochemical analysis revealed that endotoxin induced significant lipid accumulation in aged rat livers, but not those of young rats (Fig.1C). Endotoxin also affected the accumulation of FFAs in aged rat livers (Fig.1D). We also verified that lipid accumulation occurred in aged livers via histopathological analysis. As expected, obvious hepatic lipid accumulation was observed in LPS-treated aged rat livers (Fig.1E). Oil red O staining also confirmed that there was significant lipid accumulation in aged rat livers, but not the livers of young rats (Fig.1F). These results suggest that LPS induces abnormal lipid metabolism and liver lipid accumulation in aged rats.

### Aging inhibits activation of PPARα and promotes activation of SREBP1c following LPS injection

**Fig 2:**
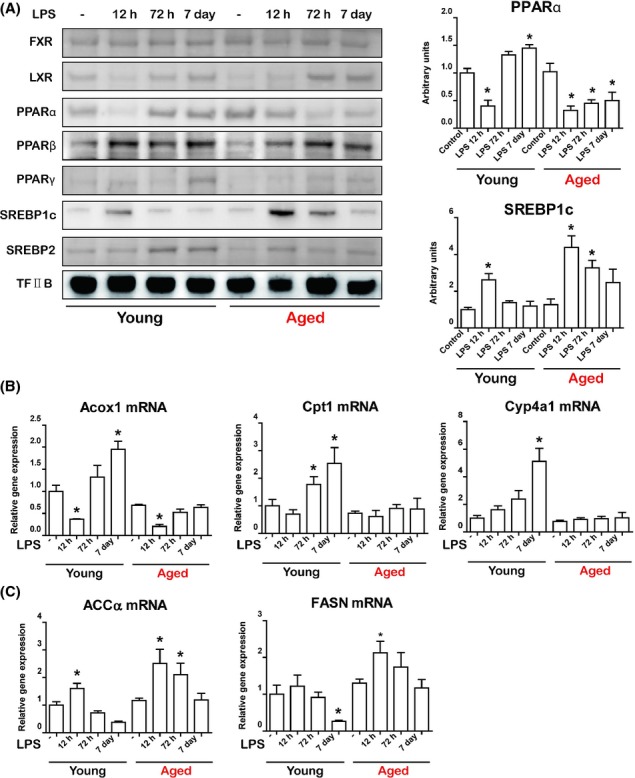
Effects of aging on lipid metabolism-associated transcription factors changes induced by lipopolysaccharide (LPS) in liver. (A) The nuclear fraction of liver homogenates was used to detect transcription factors associated with lipid metabolism. Western blots were performed to detect nuclear protein levels of FXR, LXR, PPARα, PPARβ, PPARγ, SREBP1c, and SREBP2. TF-IIB was used as control. The blots of PPARα and SREBP1c were quantified by densitometry (*n* = 5). (B) qRT–PCR of PPARα target genes (*Acox1*, *Cpt1*, and *Cyp4a1*) in LPS-treated young and aged rat livers. mRNA levels were normalized to GAPDH level. (C) qRT–PCR of SREBP1c target genes (*ACCa* and *FASN*) in LPS-treated young and aged rat livers. mRNA levels were normalized to GAPDH level. Data are expressed as the mean ± SEM. *P <* *0.05 vs. corresponding control group.

Several transcription factors are known to play pivotal roles in regulating liver lipid metabolism (Desvergne *et al*., 2006). As endotoxin provoked lipid accumulation in only aged rat livers, we next investigated whether these transcription factors were dysregulated. We examined several transcription factors that are known to play a role in regulating lipid metabolism (Desvergne *et al*., 2006). Among the many transcription factors, PPARα and SREBP1c exhibited different expression patterns between young and aged rats (Fig.2A). Nuclear localization of PPARα was significantly decreased in both young and aged rat livers 12 h after LPS injection. PPARα was recovered in young rats 72 h after LPS injection, whereas PPARα was not detected in aged rats (Fig.2A). To confirm the activity of PPARα, we assessed a well-known target gene of PPARα using qPCR. The mRNA level of *Acox1* was decreased 12 h after LPS injection in both young and aged rat livers. However, *Acox1* was detectable at significant levels in the livers of young rats but was present at lower levels in the livers of aged rats. The mRNA levels of *Cpt1* and *Cyp4a1* displayed a different expression pattern. mRNA levels of *Cpt1* and *Cyp4a1* gradually increased after LPS injection in young rat livers, but there was no increase of *Cpt1* or *Cyp4a1* in aged rat livers (Fig.2B).

In contrast to the observed decrease in nuclear PPARα during endotoxemia, SREBP1c was elevated after LPS injection in both young and aged rat livers (Fig.2A). However, nuclear SREBP1c returned to a basal level after 72 h in young rats, whereas aged rats exhibited continuous upregulation of nuclear SREBP1c (Fig.2A). As SREBP1c plays an important role in the transcription of genes associated with TG synthesis, we next examined the expression of SREBP1c target genes. mRNA expression of *ACC* and *FASN* was increased in young and aged rats a short time after LPS injection (Fig.2C). However, mRNA levels of both genes were continuously elevated in only the aged rats up to 72 h after LPS injection (Fig.2C). This suggests that LPS-induced lipid accumulation in the livers of aged rats was due to an increase in SREBP1c activity and a decrease in PPARα activity during endotoxemia.

### Inflammasome and IL-1β are upregulated in aged rat livers following LPS injection

As lipid metabolism was dysregulated in only aged rat livers following LPS injection, we next focused on elucidating the factors associated with hepatic inflammation in young and aged rats. Because LPS itself may induce changes in lipid metabolism (Feingold *et al*., 1992), we first assessed LPS clearance in the serum from young and aged rats; however, we could not detect a difference between the young and aged rats. Endotoxin levels were elevated 12 h after LPS injection, but most of the endotoxin was eliminated by the later time point (72 h) (Fig. S2A, Supporting information). Next, we focused on the major LPS receptor, TLR4, as variation in its expression may exaggerate signaling in response to LPS. However, there was no observable difference in TLR4 protein expression between the young and aged rats (Fig. S2B). Interestingly, LPS injection appeared to downregulate the expression of TLR4 in the livers of both young and aged rats (Fig. S2C).

**Fig 3:**
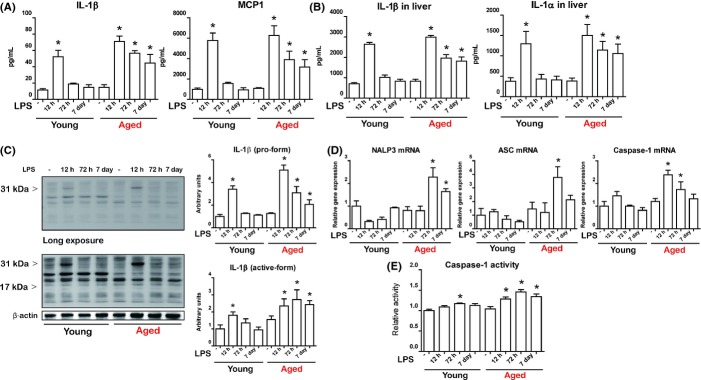
Effects of aging on lipopolysaccharide (LPS)-induced inflammasome activation and IL-1β production. (A) Measurement of serum levels of IL-1β and MCP1 following LPS injection in young and aged rats. (B) Measurement of liver levels of IL-1β and MCP1 following LPS injection in young and aged rats. (C) Pro-form (upper panel, short exposure) and cleaved form (lower panel, long exposure) of IL-1β were analyzed by Western blot. The blots of pro- and cleaved IL-1β were quantified by densitometry (*n* = 5). (D) Expression of *NALP3*, *ASC*, and *Casp-1* in the liver was measured using qPCR. (E) Caspase-1 activity was measured using a colorimetric assay. Data are expressed as the mean ± SEM. *P <* *0.05 vs. corresponding control group.

Because LPS is known to influence lipid metabolism through the induction of various cytokines (Feingold *et al*., 1992; Hardardóttir *et al*., 1994), we focused on LPS-induced cytokine changes. The serum levels of most of the cytokines exhibited an increase in the 12 h following LPS injection and returned to a basal level by 72 h in serum (Fig. S3A, Supporting information). However, IL-1β displayed a different pattern between young and aged rats (Fig.3A). While IL-1β returned to basal levels in young rats, it was significantly and continuously increased until the later time point in aged rats (Fig.3A). We also found that MCP-1, which is known to be regulated by IL-1β, also displayed a similar tendency (Fig.3A). Next, we verified the levels of cytokines in liver. Liver IL-1β also exhibited a pattern similar to that observed in serum (Fig.3B), while the other cytokines tested displayed no difference between young and aged rats (Fig. S3B). Interestingly, liver IL-1α showed same tendency with IL-1β (Fig.3B). We also confirmed changes in the protein levels of IL-1β by Western blot (Fig.3C). The livers of aged rats displayed increased levels of the premature form of IL-1β as well as increased levels of the activated form of IL-1β (Fig.3C).

IL-1β maturation and secretion is mediated by inflammasome-dependent activation of caspase-1 (Wree *et al*., 2014). qPCR analysis demonstrated that aging significantly increased expression of the inflammasome components pro-caspase-1, ASC, and NLRP3 in the liver during endotoxemia (Fig.3D). Importantly, we found increased caspase-1 activity, the effector protein of the inflammasome, in the livers of aged rats during endotoxemia compared with young rats (Fig.3E). These findings indicate that inflammasome activation and consequent IL-1β production were significantly increased in only aged rat livers during endotoxemia.

### Increased inflammation following LPS injection is only detected in aged rat livers

**Fig 4:**
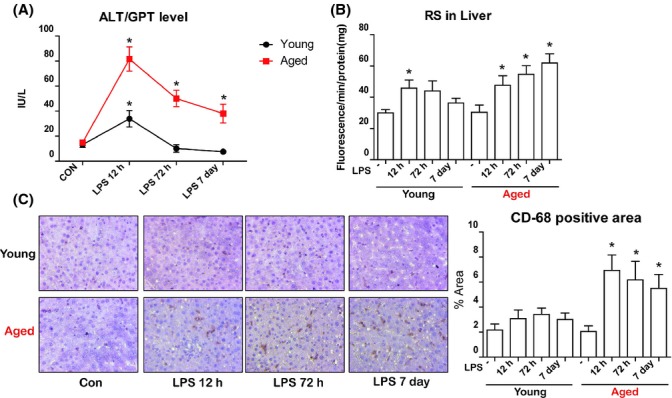
Effects of aging on lipopolysaccharide (LPS)-induced liver inflammation. (A) Liver injury was quantified by measuring serum ALT/GPT levels. (B) Oxidative stress was measured using reactive species(RS)-specific fluorescent dye. (C) Activation of liver Kupffer cells was used as a marker of liver inflammation. Immunohistochemistry was used to evaluate recruitment of CD68 positive Kupffer cells. DAB-based brown-colored region indicates the CD68^+^ Kupffer cell. Percentage of Kupffer cells was calculated. Data are expressed as the mean ± SEM. *P <* *0.05 vs. control group.

Activation of the inflammasome and the subsequent production of IL-1β are implicated in many models of liver inflammatory disease (Wree *et al*., 2014). As the inflammasome and IL-1β were significantly upregulated in aged rat livers, we next focused on endotoxin-induced inflammation differences between young and aged rats. We first measured ALT/GPT level in serum, as it is a representative marker for liver inflammation. Serum ALT/GPT levels were upregulated a short time after LPS injection (12 h) and were sustained until the later time points (72 h and 7 days) in only the aged rats (Fig.4A). Aged rat livers also exhibited continuous LPS-induced oxidative stress, which was in contrast to the livers from the young rats (Fig.4B). Kupffer cells, the resident macrophage of the liver, are known to play a pivotal role in the inflammatory response of the liver (Szabo & Csak, 2012). To determine whether activation of Kupffer cells differs between young and aged rats, we used CD-68, a reliable marker of rat macrophage-derived cells, to detect Kupffer cells using immunohistochemistry. Although LPS induced an increase in the numbers of Kupffer cells in the livers of aged rats, little increase was observed in the Kupffer cell population of young rats (Fig.4C). This suggests that increased inflammasome/IL-1β activity in the aged rat liver following LPS injection is also associated with increased liver inflammation.

### IL-1β exerts lipogenic effects on hepatocytes through the regulation of PPARα and SREBP1c

**Fig 5:**
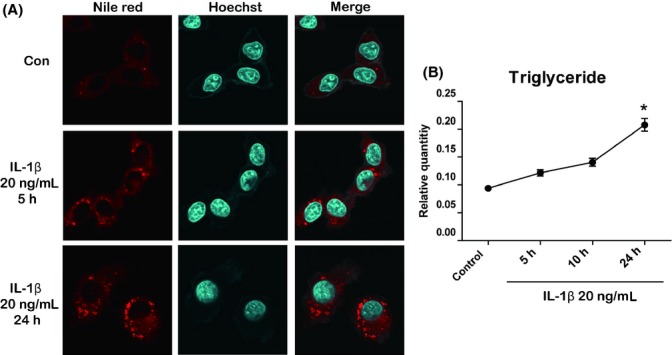
Observation of lipid accumulation in hepatic cells after treatment with IL-1β. (A) HepG2 cells were incubated with or without 20 ng mL^−1^ of IL-1β as indicated. Accumulation of lipids was visualized by Nile red staining. Cells were counterstained with Hoechst. (B) The concentration of triglycerides (TG) in HepG2 cells was measured as described in Experimental procedures. Data are expressed as the mean ± SEM. *P <* *0.05 vs. control group.

Given that activated inflammasomes and increased production of IL-1β were detected in conjunction with increased lipid accumulation and inflammation in the livers of aged rats, we further examined the mechanism of IL-1β on lipid accumulation *in vitro*. Although the direct role of IL-1β on inflammation is well known, the exact effects and mechanisms of IL-1β on changes in lipid metabolism need to be elucidated. To assess the lipogenic potential of IL-1β, we first evaluated the effect of IL-1β on lipid accumulation in HepG2 cells. We determined whether IL-1β works properly on HepG2 cells. IL-1β (20 ng mL^−1^) upregulated p65 as well as increased the nuclear localization of p65 (Fig. S4, Supporting information). Next, we examined whether IL-1β affects lipid accumulation. There was a time-dependent increase in lipid droplets in IL-1β-treated HepG2 cells as detected by Nile red staining (Fig.5A). Further quantification of intracellular TG via lipid extraction confirmed the results of Nile red staining (Fig.5B), suggesting that IL-1β directly exerts a lipogenic effect on hepatocytes.

**Fig 6:**
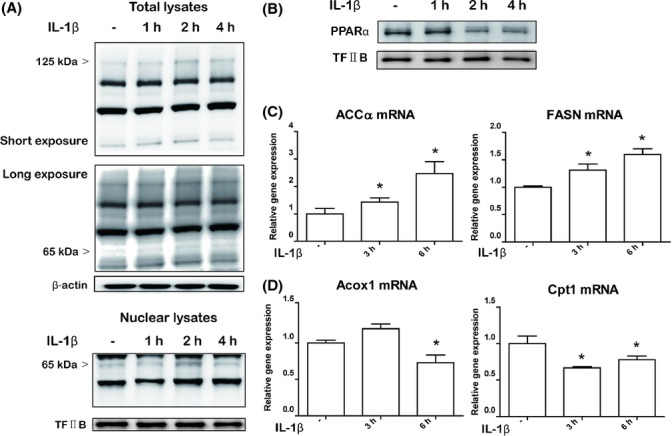
IL-1β exerts lipogenic effects on hepatocytes through the regulation of PPARα and SREBP1c transcription factors. (A) Western blot for SREBP1c from HepG2 cells treated with IL-1β. Analysis of total lysates (upper panel) reveals upregulation of total SREBP1c (short exposure) as well as cleaved form of SREBP1c (long exposure). Actin was used as control. Analysis of nuclear fractions (lower panel) reveals increased translocation of SREBP1c after IL-1β treatment in HepG2 cells. TF-IIB was used as control. (B) Western blot for PPARα from HepG2 cells treated with IL-1β. TF-IIB was used as control. (C) Expression of SREBP1c target genes (*ACCa*, *FASN*) was analyzed by qPCR. Results were normalized to GAPDH level. (D) Expression of PPARα target genes (*Cpt1*, *Acox1*) was analyzed by qPCR. Results were normalized to GAPDH level.*P < 0.05 vs. control group.

To further demonstrate the exact mechanism of IL-1β regulation of lipid accumulation *in vitro*, we investigated the changes in the levels of transcription factors associated with lipid metabolism. Treatment of IL-1β did not influence the nuclear levels of transcription factors such as FXR, LXR, SREBP2, and PPARγ (Fig. S5, Supporting information). However, IL-1β treatment significantly altered the levels of PPARα, PPARβ, and SREBP1c (Fig.6 and Fig. S5). IL-1β significantly increased the levels of the mature form of SREBP1c (125 kDa) and the cleavage form of SREBP1c (65 kDa) in total lysates (Fig.6A). IL-1β also triggered nuclear translocation of SREBP1c as determined by the detection in the nuclear fraction (Fig.6A). IL-1β also affected the nuclear form of PPARα. Treatment with IL-1β decreased the nuclear localization of PPARα by a later time point (6 h after treatment) (Fig.6B). We further checked whether the expression of SREBP1c and PPARα target genes were altered after IL-1β treatment. As expected, treatment of IL-1β significantly increased expression of SREBP1c target genes (*ACC*, *FASN*) as detected by qPCR (Fig.6C). IL-1β also decreased PPARα target genes (*Cpt1*, *Acox1*).

Taken together, these data further support our *in vivo* findings and suggest that IL-1β exerts a significant effect on hepatocyte lipid metabolism. IL-1β significantly increased the maturation of SREBP1c and decreased PPARα nuclear translocation, thereby influencing lipid accumulation in hepatocytes.

## Discussion

Aging is associated with an increase in the inflammatory response caused by various insults (Opal *et al*., 2005). Here, we show that highly activated inflammasomes and subsequent IL-1β production drove LPS-induced inflammation and lipid accumulation in the livers of aged rats. To our knowledge, this is the first report demonstrating that a low dose of endotoxin induces abnormal changes in lipid metabolites and lipid accumulation in the livers of aged rats. Furthermore, these LPS-induced effects were not observed in the livers of young rats. We further found that dysregulation of PPARα and SREBP1c is associated with increased lipid accumulation in aged rat livers. Among the many inflammation-related factors, we found that increased inflammation and lipid accumulation were associated with inflammasome activity and IL-1β production. In addition, we demonstrated that IL-1β directly regulates lipid metabolism through the modulation of SREBP1c and PPARα. Based on these findings, we have identified inflammasome and IL-1β as potential therapeutic targets in endotoxin-induced lipid accumulation associated with aging (Fig. S6, Supporting information).

Among various stresses, infection-induced inflammation is a major health issue that aged individuals face (Martin *et al*., 2006). There are many risk factors for severe infection associated with aging (Opal *et al*., 2005). However, recent works have demonstrated that enhanced innate immune responses and cytokine production against infection differentiate between the young and aged (Saito *et al*., 2003; Turnbull *et al*., 2009). Although there are many predisposing factors linking increased mortality with aging, increased inflammation is directly associated with increased morbidity as well as mortality (Saito *et al*., 2003; Opal *et al*., 2005; Turnbull *et al*., 2009). Our study also demonstrated an increased inflammatory response to endotoxin in aged rats. Through our time-dependent investigation, we identified specific inflammatory mediators that displayed an altered pattern during endotoxemia. Among the many inflammation-related factors, aged rats display highly activated inflammasomes and IL-1β production. This increase in inflammasome/IL-1β was directly connected with increased inflammation in the liver. As inflammasomes have been recently recognized as important factors in innate immunity, our findings suggest the importance of IL-1β as a specific cytokine critical in aged rats during endotoxemia.

Nonalcoholic fatty liver disease is increasingly recognized as a leading cause of liver damage and cirrhosis (Vuppalanchi & Chalasani, 2009; Tilg & Moschen, 2010). Although the exact mechanisms leading to NAFLD are not completely understood, obesity is strongly associated with NAFLD pathogenesis (Wree *et al*., 2013). In addition to obesity, chronic inflammation is an important contributing factor in NAFLD pathogenesis. As a major outer membrane constituent of gram-negative bacteria, LPS is considered a potent inducer of hepatic inflammation and lipid accumulation. LPS may be capable of stimulating inflammation, cytokine production, and accumulation of inflammatory cells within the liver (Miele *et al*., 2009; Chen *et al*., 2011). LPS also has been shown to directly stimulate hepatic fatty acid synthesis, thereby increasing hepatic TG production (Feingold *et al*., 1992). However, the molecular basis for this effect has not been clearly established because LPS injection induces numerous cytokines, including TNFα and IL-1β (Feingold *et al*., 1992; Hardardóttir *et al*., 1994). Our results demonstrate that even a low concentration of LPS exerted significant biological effects on lipid metabolism, thus supporting the role of LPS-induced inflammation in the regulation of lipid metabolism. Moreover, our results are the first to demonstrate that aged rats exhibit a different response to LPS injection, especially in changes of lipid metabolism.

Although the alterations of lipid metabolism that occur during inflammation are likely to reflect metabolic adaptations that enable the host to resist infection, uncontrolled changes might be harmful (Feingold *et al*., 1992). Indeed, our results demonstrate that in response to endotoxin, lipid metabolites were increased. However, in contrast to young rats, aged rats displayed chronically accumulated lipid metabolite levels in the serum. Because liver is known to play an important role in the regulation of lipid metabolism during stress (Reddy & Rao, 2006), we further focused on lipid metabolism in the liver. Surprisingly, we found that significant lipid accumulation occurred during endotoxemia, but only in the livers of aged rats. Because many transcription factors are known to regulate lipid metabolism during inflammation (Desvergne *et al*., 2006; Reddy & Rao, 2006), we further confirmed that PPARα and SREBP1c were dysregulated in aged rat livers. These results were intriguing because aging is associated with the increased prevalence of NAFLD (Frith *et al*., 2009; Fontana *et al*., 2013). In particular, in the presence of NAFLD, mortality is significantly increased in aged individuals. This association may result from age-related increases in obesity and diabetes, cumulative effects of lifestyle factors such as diet, or the physiological changes that are a part of the aging process. Therefore, we hypothesize that aging promotes the development of NAFLD by abnormal lipid metabolism during endotoxin-induced inflammation.

The importance of inflammasome activation in various models of NAFLD has been demonstrated recently (Szabo & Csak, 2012). Acetaminophen-induced hepatotoxicity is dependent on the activation of the inflammasome (Imaeda *et al*., 2009), and IL-1β production via activation of the inflammasome is also pivotal in hepatitis C virus infection-associated hepatic disease (Negash *et al*., 2013). Furthermore, a more recent study demonstrated a pathogenic role for inflammasome activation in alcoholic liver disease (ALD) and ischemia-induced hepatic injury (Petrasek *et al*., 2012; Kamo *et al*., 2013). In these studies, increased production of IL-1β through the activation of the inflammasome was pivotal for the pathogenesis. Our results also support the importance of inflammasome activation in liver disease. As described previously, LPS itself, or through induction of numerous cytokines, can modulate lipid metabolism in liver. However, we did not observe a difference in LPS clearance or expression of the LPS receptor (TLR4) between young and aged rats. Moreover, when we checked various cytokines in the serum and liver, most exhibited the same pattern between young and aged rats, although most of the cytokines were slightly elevated in aged rats without any significance. As IL-1β was the only cytokine that displayed a different expression pattern between young and aged rats, we further checked the activation of the inflammasome during endotoxemia. Significantly increased mRNA level of inflammasome components (NLRP3, ASC, caspase-1) was detected in aged rat livers, and the activity of caspase-1 was also significantly increased. We also observed severe inflammation in aged rats as reflected by increased serum ALT levels, increased oxidative stress, and activation of Kupffer cell. We concluded that activated inflammasomes and increased IL-1β production play a pivotal role on liver inflammation and the subsequent lipid accumulation in aged rats.

Although the role of IL-1β in hepatic inflammation is well known, the exact role of IL-1β on lipogenesis in hepatic tissue or cells is still unknown. Some studies have demonstrated a direct lipogenic role of IL-1β on hepatocytes. Ma *et al*. (2008) first demonstrated lipid accumulation after inflammatory stresses on primary hepatocytes and HepG2 cells. Petrasek *et al*. (2012) further demonstrated that even very low concentrations of IL-1β exert a significant biological effect on hepatocytes. Although they revealed the lipogenic role of IL-1β on hepatocytes, they did not determine the exact mechanism by which IL-1β influences lipid accumulation. Assuming that highly activated inflammasomes and IL-1β production directly influenced changes to lipid metabolism in aged rats, we further verified direct lipogenic mechanisms *in vitro*. We also demonstrated the lipogenic effects of IL-1β *in vitro*. IL-1β increased total SREBP1c as well as provoked cleavage and nuclear translocation. These seem to be associated with the activation of Akt signaling, which is known to be regulated by IL-1 receptor signaling, as SREBP1c can be directly regulated by Akt signaling (Yecies *et al*., 2011). We also found that IL-1β decreased nuclear levels of PPARα. Although we did not determine how IL-1β regulates the translocation of PPARα, we assume it is likely due to indirect effects because PPARα translocation occurred at a later time point (6 h after IL-1β treatment). Other transcription factors were not regulated by IL-1β treatment *in vitro*. As our *in vivo* findings demonstrated that activated inflammasomes and subsequent IL-1β production were associated with increased inflammation and lipid accumulation *in vivo*, these *in vitro* findings support our *in vivo* experiments.

In conclusion, our data demonstrate that aging increases sensitivity to endotoxin-induced liver inflammation through the activation of inflammasomes and subsequent production of IL-1β. Increased IL-1β was also associated with liver inflammation and lipid accumulation. Furthermore, we demonstrated the lipogenic role of IL-1β through the regulation of SREBP1c and PPARα. Taken together, we propose a mechanism of aging-specific vulnerability against endotoxin-induced liver inflammation and suggest a pathogenic role of the inflammasome/IL-1β pathway on liver lipid accumulation.

## Experimental procedures

### Materials

Lipopolysaccharide (*Escherichia coli* serotype O111:B5) was purchased from Sigma (St. Louis, MO, USA). IL-1β was purchased from Humanzyme (Chicago, IL, USA). The antibodies used were sourced as follows: the antibodies to LXR, FXR, PPARα, PPARβ, PAPRγ, SREBP1c, SREBP2, TLR4, β-Actin, p65, and TF-IIB were obtained from Santa Cruz Biotechnology (Dallas, TX, USA); antibodies to ED-1 (CD68) were obtained from Abcam (Cambridge, MA, USA). All other reagents were purchased from Sigma if not otherwise stated.

### Animal experiments

To investigate the effects of aging on LPS-induced inflammation and metabolism changes, male Sprague-Dawley (SD) rats aged six (young) and 24 (aged) months were used. Rats were maintained under controlled environmental conditions, a 12-h/12-h light/dark cycle and allowed *ad libitum* access to water and a standard laboratory diet. Rats were randomly divided into eight groups of five animals and acclimated 2 weeks before experiments. LPS (2 mg kg^−1^ body weight) was injected intraperitoneally in young and aged rats. Rats were euthanized 12 h, 72 h, and 7 days after injection to determine the time-dependent changes. Serum was collected for biochemical analysis. Livers were collected and either frozen immediately in liquid nitrogen for qPCR, Western blot, and other biochemical analyses, fixed in neutral-buffered formalin for histochemical examination, or frozen-fixed in OCT mounting media for Oil red O staining. All animal studies were approved by the Institutional Animal Care Committee of Pusan National University and performed in accordance with the guidelines for animal experimentation issued by Pusan National University.

### Serum biochemical analysis and cytokine measurements

Serum glucose, cholesterol, triglyceride (TG), and free fatty acids (FFAs) were analyzed using kits from Bioassay Systems (Hayward, CA, USA). To measure endotoxin clearance from serum, the LAL Chromogenic Endotoxin Quantitation Kit (Pierce Biotechnology, Rockford, IL, USA) was used. Serum alanine aminotransferase (ALT/GPT) was measured using a kit from Stanbio (Boerne, TX, USA). All the cytokines and chemokines were measured using the Luminex multiplex analysis system (Millipore, Billerica, MA, USA).

### Liver TG/FFAs measurements

To analyze hepatic TG and FFAs, liver samples were homogenized in ice-cold phosphate-buffered saline (PBS). TG and FFAs were extracted with methanol/chloroform (1:2), dried, and resuspended in 5% bovine serum albumin (BSA). The level of TG was determined using a commercially available kit from Bioassay Systems.

### Histological analysis

Livers were fixed in 10% neutral formalin, and paraffin-embedded sections were stained with hematoxylin and eosin (H&E staining). The optimal cutting temperature of frozen samples and staining with Oil red O were described previously (Petrasek *et al*., 2012). Immunohistochemistry (IHC) staining for hepatic macrophage was performed using ED-1 (CD-68, Abcam) antibody. Formalin-fixed, paraffin-embedded livers were stained using a kit (DAB-based IHC systems; Invitrogen, Carlsbad, CA, USA) according to manufacturer’s instructions.

### Protein extraction and immunoblot analysis

PRO-PREP protein extraction solution (iNtRON Biotech Inc., Gyeonggi, Korea) was used to extract total protein lysates from cells or tissues according to the manufacturer’s instructions. To extract nuclear protein, cells were washed with phosphate-buffered saline (PBS) and then with 1 mL of ice-cold PBS. Pellets were harvested at 1000 ***g*** for 5 min at 4 °C, suspended in 10 mm Tris (pH 8.0) containing 1.5 mm MgCl_2_, 1 mm DTT, 0.1% NP-40, and protease inhibitors, incubated on ice for 15 min, and centrifuged at 14 000 ***g*** for 15 min at 4 °C. Supernatants were used as cytosolic fractions and pellets were resuspended in 10 mm Tris (pH 8.0) containing 50 mm KCl, 100 mm NaCl, and protease inhibitors, incubated on ice for 30 min, and then centrifuged at 14 000 ***g*** for 30 min at 4 °C. The resultant supernatants were used as nuclear fractions. Protein concentrations were measured using a BCA assay. The samples were prepared in gel buffer (pH 6.8) [12.5 mm Tris (hydroxymethyl) aminomethane, 4% sodium dodecylsulfate (SDS), 20% glycerol, 10% 2-mercaptoethanol, and 0.2% bromo-phenol blue], at a ratio of 1:1, and were boiled for 5 min. Western blot assays were performed as described previously with minor modification (Gershoni & Palade, 1983).

### Isolation of total RNA and real-time RT–PCR

Liver RNA was purified using the TRIzol reagent (Invitrogen). RNase-free DNase-treated total RNA (2.0 μg) was reverse-transcribed with a cDNA synthesis kit from GenDEPOT (Barker, TX, USA). qPCR was performed using SYBR Green and the CFX Connect System (Bio-Rad Laboratories Inc., Hercules, CA, USA). Primer sequences and reaction conditions are shown in Supplemental Tables.

### Measurement of caspase-1 activity

Caspase-1 activity was determined in freshly prepared whole liver lysates with a colorimetric assay. The caspase-1 activity analysis was based on the cleavage of the YVAD-pNA (Ac-Tyr-Val-Ala-Asp-pNA) substrate (BioVision, Milpitas, CA, USA).

### Measurement of oxidative stress

RS generation was measured by utilizing a fluorescent probe, 2′,7′-dichlorodihydro-fluorescein diacetate (DCFDA). For tissue homogenates, 25 μm DCFDA was added to the homogenates in 50 mm phosphate buffer. Changes in fluorescence intensity were measured every 5 min for 30 min on a microplate reader (GENios; Tecan Instruments, Salzburg, Austria) with excitation and emission wavelengths of 485 and 530 nm, respectively.

### *In vitro* experiments

HepG2 cells (human hepatocellular liver carcinoma cell line) were obtained from the American Type Culture Collection (Manassas, VA, USA). Cells were cultured in Dulbecco’s Modified Eagle Medium (DMEM) containing 10% fetal bovine serum (FBS; Gibco, Grand Island, NY, USA), and penicillin/streptomycin (100 IU/50 μg mL^−1^) in a humidified atmosphere containing 5% CO_2_ in air at 37 °C. To observe lipid accumulation in HepG2 cells, cells were incubated with IL-1β for 5 h, 10 h, and 24 h. Cells were stained with Nile red dye and counterstained with Hoechst. Confocal images were obtained using a FV10i FLUOVIEW Confocal Microscope (Olympus, Tokyo, Japan). Cellular TG were measured by extracting cells with methanol/chloroform (1:2).

### Statistical analysis

Analysis of variance (ANOVA) was used to analyze differences among all groups. Differences in the means of individual groups were assessed using the Fisher’s protected least-significant difference post hoc test. Values of *P* < 0.05 were considered statistically significant.

## Supporting Information

**Fig. S1** LPS-induced changes of cholesterol and glucose in serum.

**Fig. S2** Changes of serum endotoxin level and liver TLR4 expression following LPS injection.

**Fig. S3** Effects of aging on LPS-induced cytokine production in serum and liver.

**Fig. S4** Effects of IL-1β on p65 translocation on HepG2 cells.

**Fig. S5** Effects of IL-1β on lipid metabolism-associated transcriptional factor changes.

**Fig. S6** Effects of aging on LPS-induced lipid accumulation and inflammation in liver.

**Table S1**Primers used in Real-time PCR (Rat). **Table S2** Primers used in Real-time PCR (Human).

## References

[b1] Affò S, Morales-Ibanez O, Rodrigo-Torres D, Altamirano J, Blaya D, Dapito DH, Millán C, Coll M, Caviglia JM, Arroyo V, Caballería J, Schwabe RF, Ginès P, Bataller R, Sancho-Bru P (2014). CCL20 mediates lipopolysaccharide induced liver injury and is a potential driver of inflammation and fibrosis in alcoholic hepatitis. Gut.

[b2] Carré JE, Singer M (2008). Cellular energetic metabolism in sepsis: the need for a systems approach. Biochim. Biophys. Acta.

[b3] Chen X, Zhang C, Zhao M, Shi CE, Zhu RM, Wang H, Zhao H, Wei W, Li JB, Xu DX (2011). Melatonin alleviates lipopolysaccharide-induced hepatic SREBP-1c activation and lipid accumulation in mice. J. Pineal Res.

[b4] Desvergne B, Michalik L, Wahli W (2006). Transcriptional regulation of metabolism. Physiol. Rev.

[b5] Dorshkind K, Montecino-Rodriguez E, Signer RA (2009). The ageing immune system: is it ever too old to become young again?. Nat. Rev. Immunol.

[b6] Febbraio MA (2014). Role of interleukins in obesity: implications for metabolic disease. Trends Endocrinol. Metab.

[b7] Feingold KR, Staprans I, Memon RA, Moser AH, Shigenaga JK, Doerrler W, Dinarello CA, Grunfeld C (1992). Endotoxin rapidly induces changes in lipid metabolism that produce hypertriglyceridemia: low doses stimulate hepatic triglyceride production while high doses inhibit clearance. J. Lipid Res.

[b8] Fontana L, Zhao E, Amir M, Dong H, Tanaka K, Czaja MJ (2013). Aging promotes the development of diet-induced murine steatohepatitis but not steatosis. Hepatology.

[b9] Frith J, Day CP, Henderson E, Burt AD, Newton JL (2009). Non-alcoholic fatty liver disease in older people. Gerontology.

[b10] Gershoni JM, Palade GE (1983). Protein blotting: principles and applications. Anal. Biochem.

[b11] Glass CK, Olefsky JM (2012). Inflammation and lipid signaling in the etiology of insulin resistance. Cell Metab.

[b12] Gustot T (2011). Multiple organ failure in sepsis: prognosis and role of systemic inflammatory response. Curr. Opin. Crit. Care.

[b13] Hardardóttir I, Grünfeld C, Feingold KR (1994). Effects of endotoxin and cytokines on lipid metabolism. Curr. Opin. Lipidol.

[b14] Imaeda AB, Watanabe A, Sohail MA, Mahmood S, Mohamadnejad M, Sutterwala FS, Flavell RA, Mehal WZ (2009). Acetaminophen-induced hepatotoxicity in mice is dependent on Tlr9 and the Nalp3 inflammasome. J. Clin. Invest.

[b15] Kamo N, Ke B, Ghaffari AA, Shen XD, Busuttil RW, Cheng G, Kupiec-Weglinski JW (2013). ASC/caspase-1/IL-1β signaling triggers inflammatory responses by promoting HMGB1 induction in liver ischemia/reperfusion injury. Hepatology.

[b16] Kwon HJ, Sung BK, Kim JW, Lee JH, Kim ND, Yoo MA, Kang HS, Baek HS, Bae SJ, Choi JS, Takahashi R, Goto S, Chung HY (2001). The effect of lipopolysaccharide on enhanced inflammatory process with age: modulation of NF-κB. J. Am. Aging Assoc.

[b17] Ma KL, Ruan XZ, Powis SH, Chen Y, Moorhead JF, Varghese Z (2008). Inflammatory stress exacerbates lipid accumulation in hepatic cells and fatty livers of apolipoprotein E knockout mice. Hepatology.

[b18] Martin GS, Mannino DM, Moss M (2006). The effect of age on the development and outcome of adult sepsis. Crit. Care Med.

[b19] Miele L, Valenza V, La Torre G, Montalto M, Cammarota G, Ricci R, Mascianà R, Forgione A, Gabrieli ML, Perotti G, Vecchio FM, Rapaccini G, Gasbarrini G, Day CP, Grieco A (2009). Increased intestinal permeability and tight junction alterations in nonalcoholic fatty liver disease. Hepatology.

[b20] Miura K, Kodama Y, Inokuchi S, Schnabl B, Aoyama T, Ohnishi H, Olefsky JM, Brenner DA, Seki E (2010). Toll-like receptor 9 promotes steatohepatitis by induction of interleukin-1beta in mice. Gastroenterology.

[b21] Negash AA, Ramos HJ, Crochet N, Lau DT, Doehle B, Papic N, Delker DA, Jo J, Bertoletti A, Hagedorn CH, Gale M (2013). IL-1β production through the NLRP3 inflammasome by hepatic macrophages links hepatitis C virus infection with liver inflammation and disease. PLoS Pathog.

[b22] Nesseler N, Launey Y, Aninat C, Morel F, Mallédant Y, Seguin P (2012). Clinical review: the liver in sepsis. Crit. Care.

[b23] Opal SM, Girard TD, Ely EW (2005). The immunopathogenesis of sepsis in elderly patients. Clin. Infect. Dis.

[b24] Petrasek J, Dolganiuc A, Csak T, Kurt-Jones EA, Szabo G (2011). Type I interferons protect from Toll-like receptor 9-associated liver injury and regulate IL-1 receptor antagonist in mice. Gastroenterology.

[b25] Petrasek J, Bala S, Csak T, Lippai D, Kodys K, Menashy V, Barrieau M, Min SY, Kurt-Jones EA, Szabo G (2012). IL-1 receptor antagonist ameliorates inflammasome-dependent alcoholic steatohepatitis in mice. J. Clin. Invest.

[b26] Pinheiro da Silva F, Zampieri FG, Barbeiro DF, Barbeiro HV, Goulart AC, Torggler Filho F, Velasco IT, da Cruz Neto LM, de Souza HP, Machado MC (2013). Septic shock in older people: a prospective cohort study. Immun. Ageing.

[b27] Rathinam VA, Vanaja SK, Fitzgerald KA (2012). Regulation of inflammasome signaling. Nat. Immunol.

[b28] Reddy JK, Rao MS (2006). Lipid metabolism and liver inflammation. II. Fatty liver disease and fatty acid oxidation. Am. J. Physiol. Gastrointest. Liver Physiol.

[b29] Saito H, Sherwood ER, Varma TK, Evers BM (2003). Effects of aging on mortality, hypothermia, and cytokine induction in mice with endotoxemia or sepsis. Mech. Ageing Dev.

[b30] Szabo G, Csak T (2012). Inflammasomes in liver diseases. J. Hepatol.

[b31] Tilg H, Moschen AR (2010). Evolution of inflammation in nonalcoholic fatty liver disease: the multiple parallel hits hypothesis. Hepatology.

[b32] Turnbull IR, Clark AT, Stromberg PE, Dixon DJ, Woolsey CA, Davis CG, Hotchkiss RS, Buchman TG, Coopersmith CM (2009). Effects of aging on the immunopathologic response to sepsis. Crit. Care Med.

[b33] Vuppalanchi R, Chalasani N (2009). Non-alcoholic fatty liver disease and non-alcoholic steatohepatitis. Hepatology.

[b34] Wree A, Broderick L, Canbay A, Hoffman HM, Feldstein AE (2013). From NAFLD to NASH to cirrhosis-new insights into disease mechanisms. Nat. Rev. Gastroenterol. Hepatol.

[b35] Wree A, Eguchi A, McGeough MD, Pena CA, Johnson CD, Canbay A, Hoffman HM, Feldstein AE (2014). NLRP3 inflammasome activation results in hepatocyte pyroptosis, liver inflammation, and fibrosis in mice. Hepatology.

[b36] Yecies JL, Zhang HH, Menon S, Liu S, Yecies D, Lipovsky AI, Gorgun C, Kwiatkowski DJ, Hotamisligil GS, Lee CH, Manning BD (2011). Akt stimulates hepatic SREBP1c and lipogenesis through parallel mTORC1-dependent and independent pathways. Cell Metab.

