# Supplementary figures and images for: Age-related sensitivity to endotoxin-induced liver inflammation: Implication of inflammasome/IL-1β for steatohepatitis
Source: Aging Cell. 2015 Apr 7;14(4):524–33. doi: 10.1111/acel.12305 (PMC4531067; doi:10.1111/acel.12305)

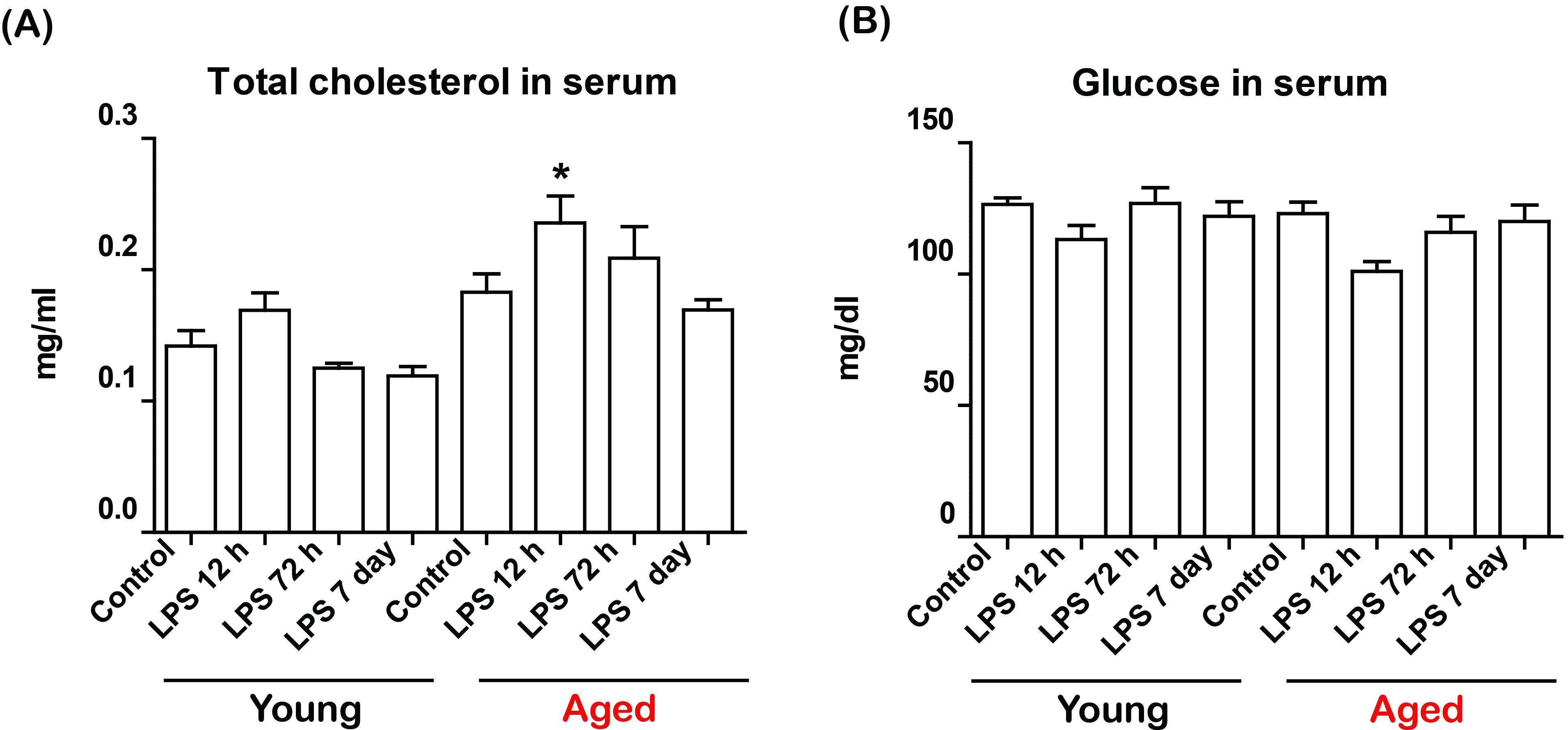

Supplement: Supplementary file 1 [file acel0014-0524-sd1.jpg]

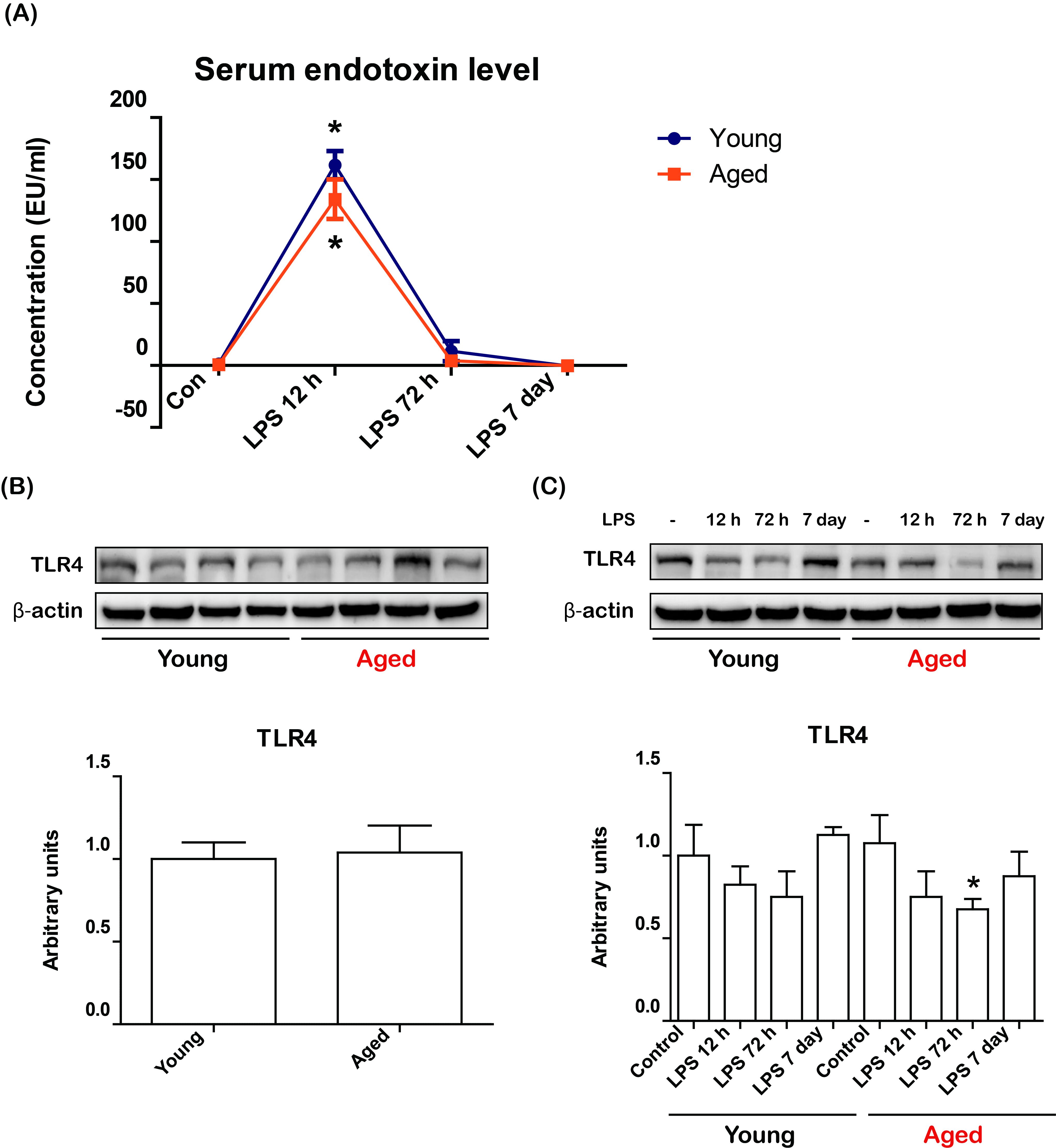

Supplement: Supplementary file 2 [file acel0014-0524-sd2.jpg]

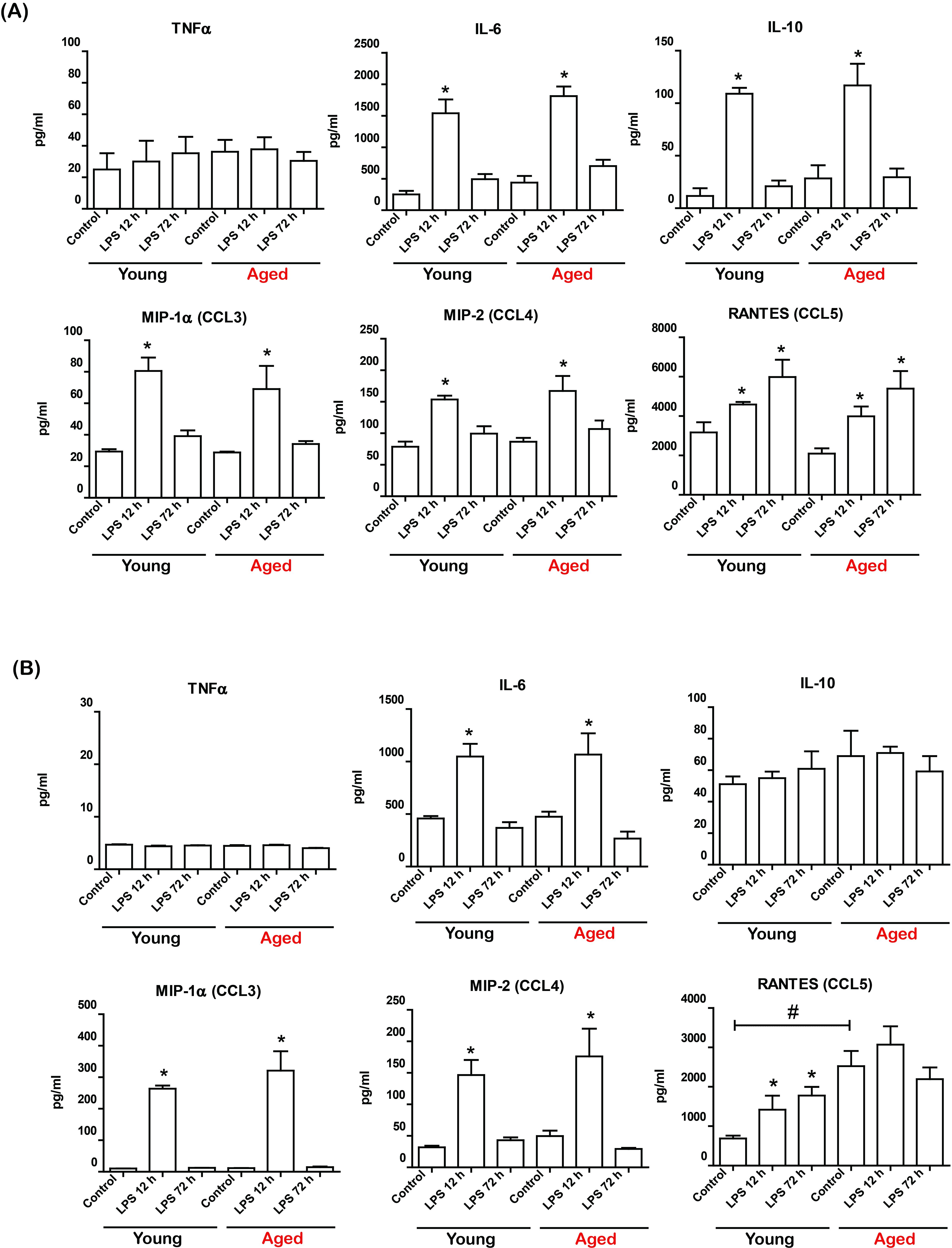

Supplement: Supplementary file 3 [file acel0014-0524-sd3.jpg]

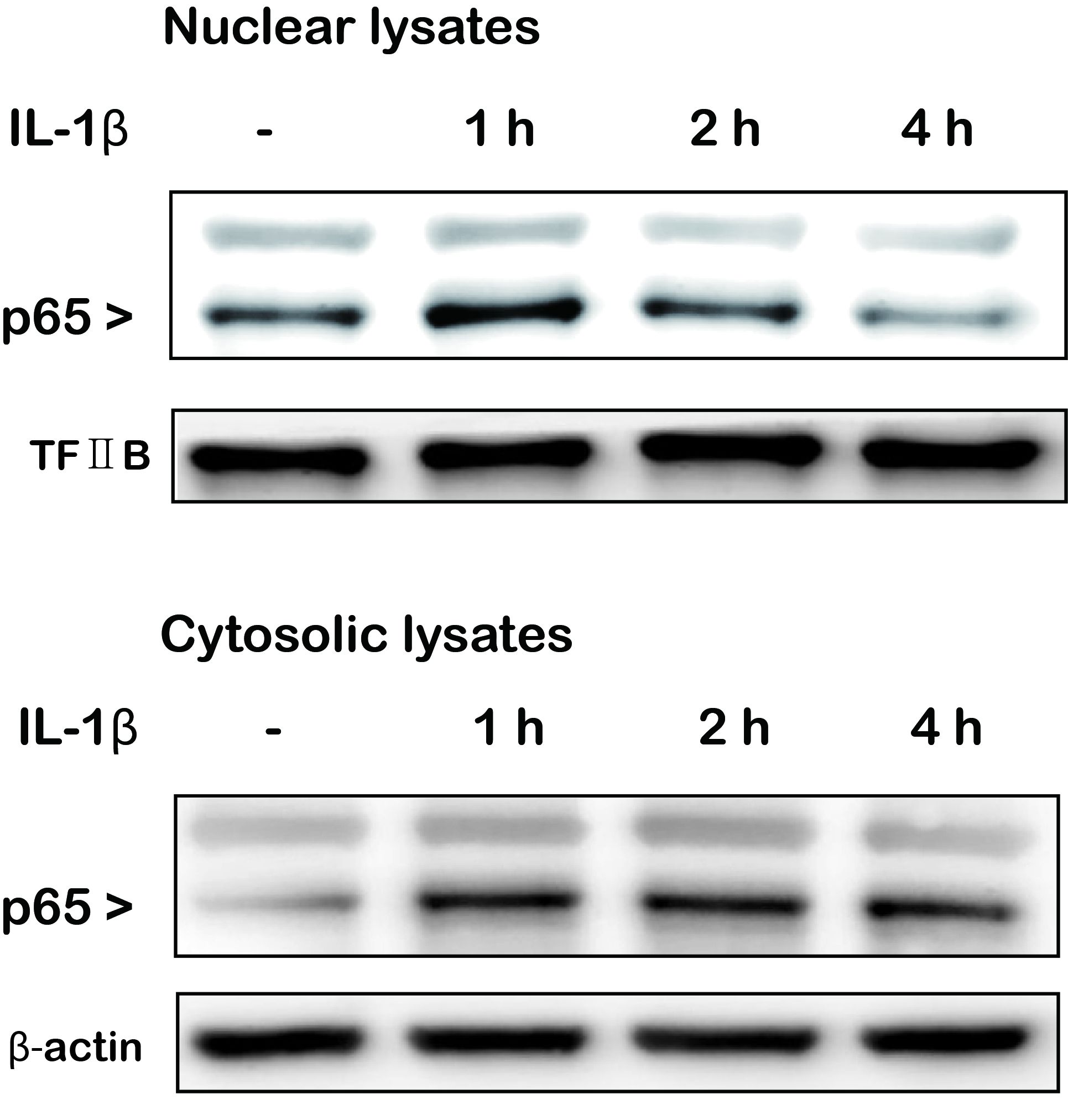

Supplement: Supplementary file 4 [file acel0014-0524-sd4.jpg]

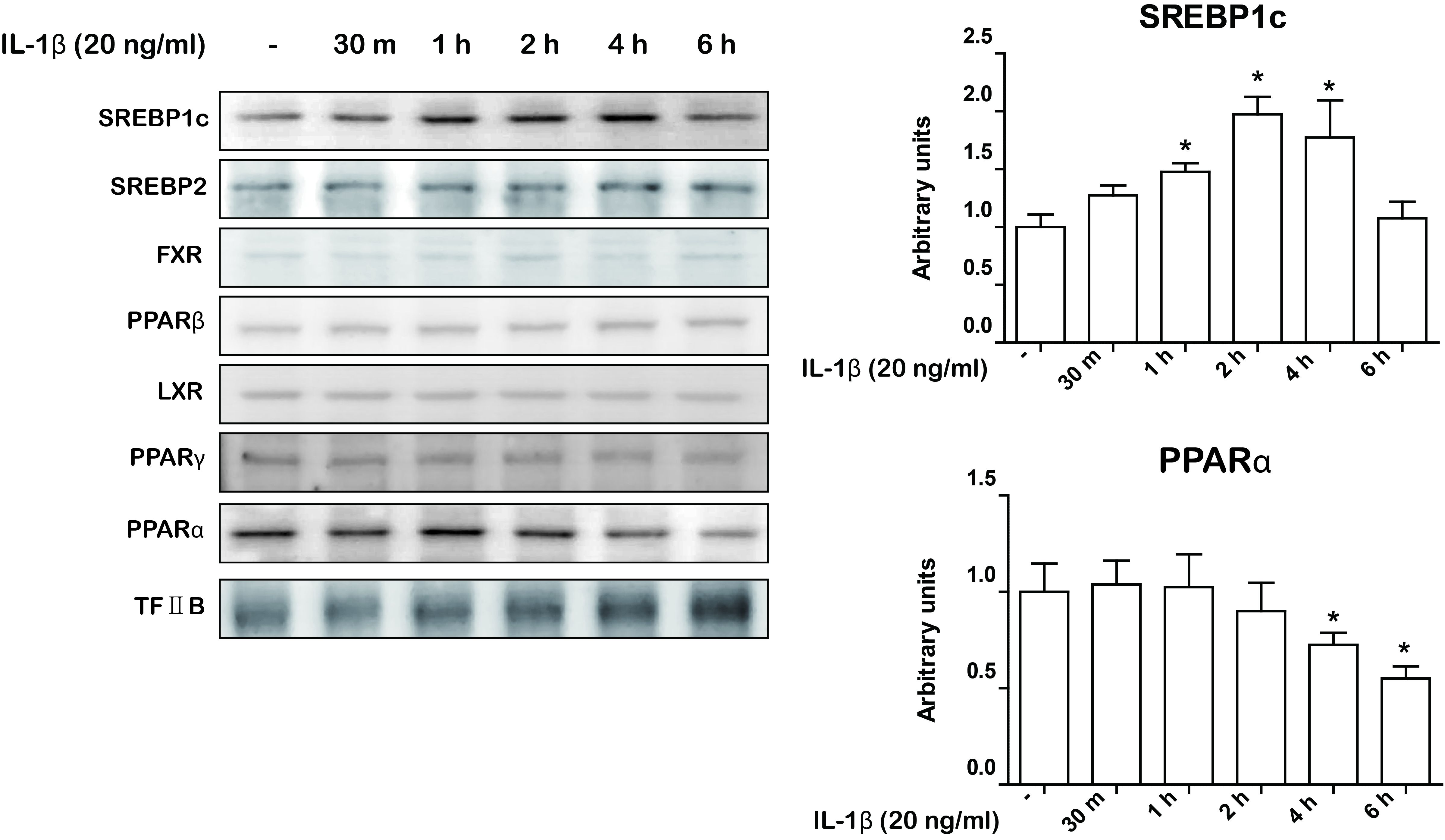

Supplement: Supplementary file 5 [file acel0014-0524-sd5.jpg]

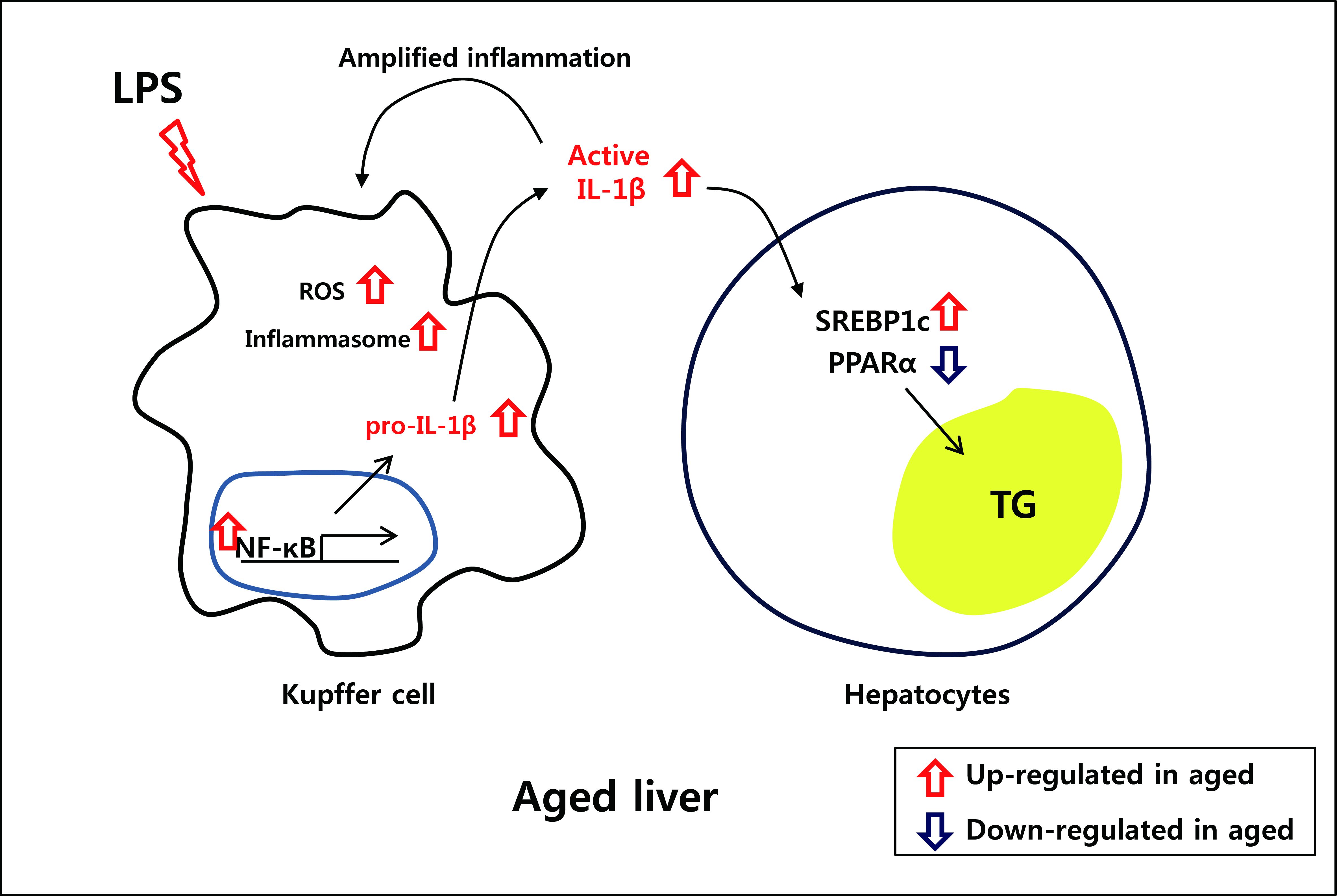

Supplement: Supplementary file 6 [file acel0014-0524-sd6.jpg]
