# Supplementary material for: Age-related sensitivity to endotoxin-induced liver inflammation: Implication of inflammasome/IL-1β for steatohepatitis
Source: Aging Cell. 2015 Apr 7;14(4):524–33. doi: 10.1111/acel.12305 (PMC4531067; doi:10.1111/acel.12305)
Supplement: Supplementary file 7 [file acel0014-0524-sd7.docx]

**Supplementary tables**

**Supplementary table1. Primers used in Real-time PCR (Rat)**

| Genes | Forward(5’-3’) | Reverse(5’-3’) |
| --- | --- | --- |
| Casp1 | ACC GAG TGG TTC CCT CAA GT | CCT GCA GCA GCA ACT TCA TT |
| NLRP3 | ACC GAC GTC TCT GCT TTC CT | AGG ACC TTC ACG TCT CGG TT |
| ASC | CAT TGC CAG GGT CAC AAA AG | CCA AGT AGG GCT GTG TTT GC |
| Acox1 | TCA GCA GGA GAA ATG GAT GC | TGG AAG TTT TCC CAA GTC CC |
| Cpt1 | AAG CTG TGG CCT TCC AGT TC | GGA TGA AAT CAC ACC CAC CA |
| Cyp4a1 | ACC TGT TCC AGG CAT TGT CA | AGA AGG GCA GGA ATG AGT GG |
| ACC | GGC ACT CTG ATC TGG TCA CG | GCT CCG CAC AGA TTC TTC AA |
| FASN | AGT GAG TGT ACG GGA GGG CT | GCT GGG ACA CAT GTG ATG GT |

**Supplementary table2. Primers used in Real-time PCR (Human)**

| Genes | Forward(5’-3’) | Reverse(5’-3’) |
| --- | --- | --- |
| Acox1 | TTA CCC AGC CCT GGC TTA AT | AGG TCA CAG CTG TCC AAC CA |
| Cpt1 | GTG CTC TGA GGC CTT TGT CA | GGT CCA GGT AGA GCT CAG GC |
| ACC | AGG TAG TTG TTG GCC TGC CT | TCA CCA CCT GTG GAA CTG CT |
| FASN | GAC GTC TGC AAG CCC AAG TA | CAT CGT CTC CAC CAA AAT GC |
